# Ergonomic Evaluation of Biomechanical Hand Function

**DOI:** 10.1016/j.shaw.2014.09.002

**Published:** 2014-09-22

**Authors:** Kyung-Sun Lee, Myung-Chul Jung

**Affiliations:** Department of Industrial Engineering, Ajou University, Suwon, Korea

**Keywords:** anthropometry, electromyography, hand function, kinematics, kinetics

## Abstract

The human hand is a complex structure that performs various functions for activities of daily living and occupations. This paper presents a literature review on the methodologies used to evaluate hand functions from a biomechanics standpoint, including anthropometry, kinematics, kinetics, and electromyography (EMG). Anthropometry describes the dimensions and measurements of the hand. Kinematics includes hand movements and the range of motion of finger joints. Kinetics includes hand models for tendon and joint force analysis. EMG is used on hand muscles associated with hand functions and with signal-processing technology.

## 1 Introduction

The human hand is composed of a thumb, index finger, middle finger, ring finger, little finger, and palm, which includes the thenar eminence, the hypo thenar eminence, and creases. The fingers contain 19 bones of distal phalanges, middle phalanges, and proximal phalanges, and metacarpal bones. Thus, the fingers have metacarpophalangeal (MCP), proximal interphalangeal (PIP), and distal interphalangeal (DIP) joints, whereas the thumb has carpometacarpal (CMC), MCP, and interphalangeal (IP) joints. The wrist contains the following eight bones: the hamate, pisiform, triquetral, capitate, lunate, trapezoid, trapezium, and scaphoid [1]. In total, the hand has 27 bones and 28 muscles [2]. These numerous bones and muscles enable the hand to perform various functions.

The hand is frequently used in activities of daily living and industrial fields because of its many functions. This can cause numerous musculoskeletal disorders (MSDs) in the hand relative to the lower limbs, such as De Quervain's tenosynovitis, trigger finger, ganglionic cysts, hand–arm vibration syndrome, and BlackBerry thumb [3]. Hand disorders account for one third of all injuries at work, one fourth of lost work time, and one fifth of permanent disabilities [4]. Hand discomfort and injuries occur when a task requires a hand strength that exceeds the worker's capability, an awkward posture, and/or repetitive motion. Individuals with hand MSDs are limited in their activities due to their reduced grip strength and ability [5–8].

The handgrip is an important and basic function for various movements. Object manipulation with a stable handgrip is one of the most frequent movements performed in activities of daily living and occupational fields. A reduction in the grip strength and control ability can be attributed to physical and psychosocial factors. Physical factors can include a reduction in the number of contracting muscle fingers, reduction in the firing rate of motor units, and change in the muscle fiber type. Psychosocial factors can include pain, a fear of pain, and a fear of reinjury [8]. Pain can reduce the grip force, which decreases voluntary muscle activity. This manifests as decreases in the force generation, electromyographic (EMG) activity [9–11], motor unit discharge rate [12], and ability to maintain a grip force [13,14]. MSDs can cause a person's physical and psychological capacities to deteriorate.

Many researchers in the ergonomics field have been trying to understand how humans use their hands and which factors affect the hand-function capacity. In particular, the physical capacity of the hand has typically been evaluated by biomechanical methodologies. Biomechanical analysis of the human hand can be divided into anthropometry, kinematics, kinetics, and EMG [15].The application of biomechanical principles is important for preventing MSDs in order to improve working conditions and performance. In ergonomics, safety, and health, the hand is mainly evaluated to reduce the risk of MSDs. In product development, the hand is actively studied for the design of hand tools and cell phones. In rehabilitation, the hand is studied to evaluate the difference between patients and healthy individuals. Studying the hand is important for the development of hand-related simulations and robots in the digital manufacturing simulation and intelligence robot fields.

Detailed information on the technologies and methodologies used for hand analysis is required for nonexperts in the field of biomechanics such as hand-tool designers and safety supervisors to understand and choose easy and suitable methods. Hand anthropometry is simply the basis of biomechanical analysis. The range of motion (ROM) is the most commonly used functional measurement variable. Anatomical measurements and the ROM are usually used to design hand products and rehabilitation. The three-dimensional (3D) motion analysis system is currently the most commonly used technique to measure kinematic variables such as the trajectory, angle, velocity, and acceleration. This system needs marker sets and kinematic models for analysis. Several kinds of marker sets and kinematic models have been developed based on the purposes of different studies, and the accuracy of the system has been improved. Thus, it can provide important information for researchers to choose a suitable method. Kinetic hand models have been developed for analyzing the internal load (force and moment) of tendons and muscles during static and dynamic motions. These kinetic hand models have advantages and disadvantages with regard to the measurement method and complexity. Information from kinetic hand models can help a researcher design an experiment design. EMG is most commonly used in various research fields to evaluate the muscle activity, fatigue, and conduction velocity. For accurate analysis, understanding the use of the EMG equipment, electrode placement, muscle position, and signal-processing methods is important.

This paper presents a literature review of some technologies and methodologies used for hand-function analysis based on a biomechanical approach and the results of previous studies related to hand functions. The following four categories of hand-function analysis are covered: (1) anthropometry, (2) kinematics, (3) kinetics, and (4) EMG.

## 2 Methods

For this review, a systematic search was conducted using PubMed, Elsevier Science, and ScienceDirect databases, and Google Scholar on studies published from 1960 to 2014. The search was restricted to papers published in English and containing the terms “hand biomechanics,” “hand function,” “hand anthropometry,” “hand kinematic,” “hand kinetic,” “EMG of hand,” “finger joint angle,” “finger tendon force,” or “biomechanical hand model” in the title, abstract, or keywords. The initial search of the database yielded about 450 results. After a review of the titles and abstracts to reject duplicated articles, 245 articles were selected. After applying inclusion and exclusion criteria, 19 articles related to hand anthropometry were selected, and 31 articles related to hand kinematics were identified from the manual targeted search. Eighteen articles or books related to hand kinetics, 10 articles related to hand EMG, and 26 articles related to hand anatomy, MSDs, posture, and functions were selected. In total, 104 articles were selected for inclusion in the current review (including 6 books and 6 reports). In the following sections, the term “reviewed articles” refers to the 104 selected articles.

## 3 Hand anthropometry

### 3.1 Technology for hand anthropometry evaluation

Hand anthropometry is important to the design of products for human hands. Examples include machine guards, hand tools, and luggage handles. Hand anthropometric parameters are categorized into anatomical measurement variables such as the length, width, and circumference [16–18]; functional measurement variables such as the handgrip span, flexion and extension ROMs of the fingers and wrist, and abduction/adduction and deviation ROMs of the wrist in engineering anthropometry [16,18–20].

Hand anthropometry can be directly measured using digital calipers, circumference tapes, and finger circumference gauges [16,21] and can also be measured from photographs [18,22] and scans [23,24]. Goniometers and 3D motion analysis systems are used to measure the width, flexion, and extension ROMs [25]. Direct measurement is easy and efficient, but skin movement and experimenter error can occur. Photography measurement requires less time than direct measurement, and the recorded information can be repeatedly used [26], but measuring the circumference is difficult. Although 3D scans can be used to measure diverse hand areas precisely, data can be distorted due to movements during the scan.

### 3.2 Anatomical measurement variables

In general, anthropometry for anatomical measurement variables is divided into general and application surveys. General surveys are used to explain the hand variation of large populations. Their main purpose is to describe populations. By contrast, application surveys are used to gather data for a specific product. Therefore, an application survey often uses few individuals but with strictly defined populations such as occupational groups [18].

Following the trend of general surveys for hand anthropometry, Vicinus [27] measured 44 dimensions of both hands in 253 males. The results for the left and right hands were significantly different. The left hand had a larger breadth than the right hand, whereas the right hand had a larger length than the left hand. Moreover, the correlation between the hand length and breadth dimensions was generally poor. Garrett [28,29] conducted a comprehensive general survey on 148 males and 211 females to measure 34 dimensions of the hand and 17 dimensions of engineering anthropometry [16]. This study showed a wider range of hand dimensions than previous studies. Gooderson et al [30] measured 62 dimensions of the left and right hands in 300 males and 187 females in the British army. Similar to Vicinus [27], they found a low correlation between the hand length and breadth dimensions. Greiner [18] measured 64 hand dimensions. Recently, Okunribido [31] measured 18 dimensions of the hand in 37 females from Ibadan and western Nigeria and compared them with those of other populations. The results showed that hand dimensions differed between populations. Similarly, Mandahawi et al [32] measured 24 hand dimensions in 115 males and 120 females and analyzed the difference between sexes and between Jordanians and other populations. Their results showed significant differences with regard to the sex and population.

With regard to examples of application surveys for hand anthropometry, Barter and Alexander [33] measured 18 hand dimensions in 100 individuals to develop a glove sizing system. In their study, hand dimensions were selected for developing the glove system, and these dimensions are not normally measured in most hand surveys. Rosenblad-Wallin [34] measured 33 hand dimensions for the development and design of army gloves.

Hand anthropometry data are used to design ergonomic tools or equipment and space. Thus, the measurement criteria and dimensions of hand anthropometry differ according to the study purpose, such as general and application surveys. Although the hand dimensions measured in application surveys were focused on specific user groups, they were part of those considered in general surveys. Table 1 presents the hand anthropometry dimensions for the previous studies summarized in this literature review. The dimensions in Table 1 represent the length, breadth, and circumference, which are commonly used as basic data in hand anthropometry.

## 4 Hand kinematics

### 4.1 Technology for hand kinematics

Numerous studies have evaluated the angle, velocity, trajectory, and acceleration during various hand functions. The following are common devices used for measuring various hand functions: X-rays, magnetic resonance imaging (MRI), manual goniometers, electrogoniometry, video technique, and marker-based motion analysis systems [35–41]. X-ray and MRI analyses are common methods for clinical observation. However, X-ray measurements carry the risk of radiation exposure [35]. The thumb trapeziometacarpal joint is difficult to measure with goniometry [42]. To compensate for these limitations, current research is actively studying the use of motion analysis systems for measuring hand functions. Motion analysis systems analyze the posture and movement continuously by calculating the 3D trajectories and have the benefit of obtaining more reliable data than other methods [41,43–45]. A motion analysis system requires reflective markers to adhere to hand joints for measurement; the angle, velocity, trajectory, and acceleration of each joint are then evaluated using a model based on a mathematical algorithm.

Four types of marker sets can be used for hand analysis. There are three skin-marker attachment methods. The “one marker per joint” attachment method attaches markers to each finger joint head [41,46–51]. The “two markers per segment” method attaches markers to the distal and proximal heads of the finger segments [52–55], and the “three markers per segment” method attaches markers with a triangular shape to finger segments [56,57]. The “one marker per joint” attachment method has been used to analyze static conditions such as power and pinch grips. The “two markers per segment” attachment method has been used to analyze dynamic movements such as a pinching motion or the ROM of finger joints. The “three markers per segment” attachment method has been used to analyze dynamic movements such as a gripping motion or the ROM of finger joints. The “cluster marker” attachment method has been used to measure the ROM of finger joints [58,59]. The “one marker per joint” attachment method has been recommended for use in the clinical research field because it causes less discomfort to patients when they move their hand, and it is easy to use the same marker placement for each patient. The “two markers per segment”, “three markers per segment”, and “cluster marker” attachment methods have been recommended for use in the biomechanical field because they are less affected by skin movement.

The Eulerian angle model [25,53,60,61] and Cheng and Pearcy's model [62] are commonly used to analyze the angle, velocity, trajectory, and acceleration of a motion based on the measured markers. The Eulerian angle model is the most commonly used model for motion analysis and explains the orientation of a rigid body in space. An arbitrary direction in space is obtained by three rotations using Eulerian angles. From this, the finger joint flexion/extension, abduction/adduction, and supination/pronation are calculated. Thus, each model uses different mathematical algorithms. The velocity, trajectory, and acceleration are also calculated from this model. The Eulerian angle model can calculate the rotation angle, whereas Cheng and Pearcy's model cannot. However, the Eulerian angle model can overestimate or misinterpret the flexion/extension and abduction/adduction angles based on 3D joint rotations [62].

Table 2 lists the marker attachment methods and models used in previous studies. The calculated angles differ according to the kinematic model and marker attachment method. Cheng and Pearcy's model uses the one marker per joint and two markers per segment attachment methods for hand analysis. This model and the marker attachment methods can only calculate the angle of 2D planes because a 3D axis cannot be defined with only one or two markers. By contrast, an Eulerian angle model with the three markers per segment and cluster marker attachment methods can be used to calculate the angles of all dimensional planes.

### 4.2 ROM of hand

The ROM of the hand is the most commonly used functional measurement variable. The ROM measurements include the flexion/extension, abduction/adduction, and pronation/supination of the CMC, MCP, and IP joints of the thumb, and MCP, PIP, and DIP joints of the other four fingers [25]. Finger motion measurements are divided into active ROM (AROM) and passive ROM (PROM) [64]. Similarly, Hume et al [65] classified their finger motion measurements as functional ROM (FROM) and normal ROM (NROM). PROM and NROM take the maximum and minimum angles in static positions, whereas AROM and FROM explain dynamic or functional movements such as gripping or pinching. Chao et al [25] examined the FROM for the fingertips during pinching and grasping, and Hume et al [65] examined the FROM for various activities of daily life. Table 3 presents the ROM for hand flexion [66].

Table 3 also provides the flexion ROM of each finger joint for the previous studies summarized in this literature review. The angle difference in each study differed according to the AROM and PROM. The angle of the MCP joint showed the largest variation in the previous studies.

Chao et al [25] presented joint flexion angles for the index finger in a variety of hand functions, which include the basic pinch and grasp, flexion/extension, radial/ulnar deviation, abduction/adduction with the middle finger, and several common activities of daily living (Table 4). Detailed angle data on various hand functions are needed for biomechanical analysis. The joint force, tendon force, moment, and torque can be calculated from the detailed angle data through inverse dynamic methods.

## 5 Hand kinetics

### 5.1 Technology for kinetics evaluation

Studies on hand kinetics have analyzed the force, moment, and torque of the fingers and tendons. These studies have used a tendon-force-measurement system [66], force transducers [71], dynamometers [72], force gloves [73], and pinch gauges [74] to take measurements. The tendon forces from the extrinsic muscles of the hand have been measured directly by instrumenting the tendon [75–77].

Excluding direct measurements, models have been developed to predict finger muscle or tendon forces during isometric hand functions [25,60,78–81], identify the characteristics of hand movements during grasping motions [82], and estimate the fingertip location and muscle excursion from measured finger poses [83]. In ergonomics, most kinetic models of the hand are based on Landsmeer's [84] tendon pulley model to identify finger movements in various hand postures and predict finger muscle and tendon forces under 2D static conditions.

### 5.2 Kinetic hand model

There are two common methods for analyzing tendon forces, namely, (1) analytical models and (2) experimental direct tendon-force-measurement models. Analytical models are based on the equation of static equilibrium at each joint of the finger to evaluate the tendon forces based on an externally applied force. Analytical models have a problem when the system being analyzed is redundant (i.e., there are more muscles than strictly necessary to obtain equilibrium across a joint). To solve this problem, two methods have been used, namely, reduction [60,85] and optimization [86]. The reduction method is used to reduce the number of excessive variables until the number of unknown forces is equal to the number of required equilibrium equations, thus eliminating static indeterminacy. In contrast to eliminating unknown muscle forces in the redundant equation system, the optimization method involves obtaining a unique solution from a mathematical formulation and optimization algorithm.

Experimental direct tendon-force-measurement models provide a more comprehensive understanding of the mechanism of the tendons inside the fingers. There are three common methods for experimental analysis, namely, (1) EMG, (2) *in vivo*, and (3) cadaveric. The EMG method is a readily available technique that can be applied to force and muscle-function analyses. For *in vivo* methods, many researchers have developed force transducers to directly measure the tendon force during various hand functions [75,76,87,88]. In case of cadaver study, the mass, volume, and muscle fiber length are measured to estimate the tendon force during hand function from cadaver [78,87]. Table 5 lists the tendon and joint forces in the flexor digitorum profundus (FDP), flexor digitorum superficialis (FDS), MCP, PIP, and DIP for various hand functions. Many researchers have attempted to gather accurate data on internal loads during various hand functions because they can be used to evaluate physical loads. The forces of the tendons and joints differ according to the hand functions and fingers. In general, the MCP joint of the index finger exhibits the largest joint force for various hand functions. However, previous studies have provided insufficiently accurate data for all finger joints.

## 6 Hand EMG

### 6.1 Hand muscles and technology of surface EMG

Surface EMG (sEMG) can be used to evaluate various biomechanical characteristics, including localized muscle activity, fatigue, and conduction velocity [91]. The musculoskeletal system conducts the motor unit active potential, which can be expressed as the firing rate in sarcolemma. The firing rate is the standard used for evaluating muscle activity based on the signal amplitude. EMG provides a physiological method for assessing muscle usage and the magnitude of muscular loading and is directly related to muscular effort [92]. Muscle activity during different occupational activities is often evaluated by EMG and presented in terms of the percentage of maximal activity [93]. Christensen [94] defined muscle fatigue as any reduction in the force-producing capacity of a muscle.

The muscles associated with hand functions can be divided into extrinsic and intrinsic muscle groups. Extrinsic muscles originate in the forearm and are generally larger; they generate most of the force of the hand. Intrinsic muscles are entirely contained in the hand and are smaller; they are associated with fine finger movement. The extrinsic muscles of the hand can be divided into the following two groups based on location: anterior and posterior. Each muscle group can be further classified as superficial and deep. The anterior muscles serve as flexors, and the posterior muscles serve as extensors.

In ergonomics studies, two types of electrodes are used to record EMG signals. The sEMG techniques are much more common unless there is a specific justification for using the fine-wire method. sEMG represents the activity of individual muscles or muscle groups over which the electrodes are placed. Because small and deep muscles are more difficult to record with sEMG, there is an increasing interest in recording the activity of larger muscles or muscle groups. sEMG for noninvasive assessment of muscles has recommendations for (1) sEMG sensors and sensor placement, (2) sEMG signal processing, and (3) sEMG modeling. Table 6 lists the hand muscles and the origins, insertions, and locations of common extrinsic muscles for hand functions [95–97]. These muscles are most commonly used to analyze and record various hand functions. The FDS, FDP, and flexor pollicis longus are the major muscles for the flexion and extension of four fingers. The extensor pollicis longus, extensor pollicis brevis, extensor digitorum communis, and abductor pollicis longus are the major muscles for the flexion, extension, and abduction of the thumb. These muscles are located in the forearm and are among the largest hand muscles; thus, sEMG is suitable for use.

### 6.2 Signal-processing technology for EMG evaluation

To evaluate the amplitude of an EMG signal, many signal-processing methods have been suggested, such as the mean absolute value, root mean square (RMS), envelope detection, and ensemble averaging [98]. During maximal voluntary contraction (MVC), several changes are observed. The integrated EMG or RMS shows a gradual decrease. The mean power frequency (MPF) shows a rapid shift to a lower frequency during sustained MVC [99].

Previous researchers have used EMG to study the mechanism of intrinsic and extrinsic finger muscles during specific hand positions and power grips. Armstrong et al [3] used rectified sEMG signals from the forearm flexor muscles to predict finger forces produced during tasks involving pinching, grasping, and pressing. Researchers have continued to examine the feasibility of predicting the grip force from EMG data with reasonably good results [100,101].

## 7 Discussion

This paper presents a literature review of some technologies and methodologies used for hand-function analysis based on a biomechanical approach. Four approaches to hand-function analysis are presented, namely, (1) anthropometry, (2) kinematics, (3) kinetics, and (4) EMG. Anthropometry includes technology to evaluate hand-measurement variables. Kinematics includes technology to evaluate the ROM of each finger joint. Kinetics includes technology and various kinetic hand models for the analysis of tendon and joint forces. EMG includes hand muscles associated with hand functions, sEMG technology, and signal-processing technology.

In general, anatomical measurement variables are classified for use in general or application surveys based on the purpose of the study. A general survey measures a large number of hand dimensions of numerous individuals; its main purpose is to describe a population. By contrast, an application survey measures fewer hand dimensions because only variables that are closely related to the product of concern are selected and measured. Thus, the measured dimensions and number of individuals vary depending on the purpose. However, no sufficient studies were performed to standardize the optimal number of individuals and related dimensions. Therefore, a general survey of hand anthropometry is required to determine the optimal number of individuals that provides reliable statistic data. Application surveys of hand anthropometry should be used to develop standards for dimensions closely related to the product of concern.

In kinematics, hand-function analysis uses various marker sets and models to evaluate the angle, velocity, trajectory, and acceleration of the hand. Techniques for measuring various hand functions include X-rays, MRI, manual goniometers, electrogoniometry, video techniques, and marker-based motion analysis systems. To analyze the angle, velocity, trajectory, and acceleration of a hand based on the measured marker, the Eulerian angle model and Cheng and Pearcy's [62] model are commonly used. Finger motion measurements are roughly classified into AROM, PROM, NROM, and FROM. PROM and NROM measure the maximum and minimum angles in static positions, whereas AROM and FROM explain dynamic or functional movements such as gripping or pinching.

Studies on kinetics have analyzed the force, moment, and torque of fingers and tendons. These parameters can be measured either directly or indirectly. Equipment used for measurement includes tendon-force-measurement systems, novel force transducers, dynamometers, force gloves, and pinch gauges. Tendon forces from the extrinsic muscles of the hand are measured directly by instrumenting the tendon. Kinetic hand models can be divided into analytical and experimental direct tendon-force-measurement models. Analytical models are based on the equation of static equilibrium at each joint of the finger and such models evaluate the tendon force based on an externally applied force. Experimental direct tendon-force-measurement models provide a more comprehensive understanding of the mechanism of the tendons inside the fingers.

The muscles associated with hand functions can be divided into extrinsic and intrinsic muscle groups. Six extrinsic muscles are commonly monitored in hand-function analysis using sEMG. Table 6 lists the action, origin, insertion, and location of these muscles. Most studies have used signal-processing techniques such as zero crossing, RMS, average EMG amplitude, mean %EMG, MPF, and maximal voluntary electrical activity. Previous studies on muscle fatigue and characteristics based on the EMG signals have only considered static postures, not dynamic postures, and simply considered the relative muscle activity from the MVC. However, in the case of dynamic gripping tasks, the muscle fiber depth and length change with time, and the distance between the sEMG electrode and muscle fiber also changes. A muscle–tendon moment difference is generated with changes in the muscle contraction velocity, and rapid motor unit recruitment by contraction shows flexible signal characteristics [102]. Therefore, using EMG on dynamic contractions requires a different interpretation from static contractions.

The biomechanical analysis of the hand is an interdisciplinary study of the mechanical movement and force of the hand's musculoskeletal system; it includes hand anthropometry, kinematics, kinetics, and EMG. Biomechanical analysis aims to provide design guidelines for hand tools and devices or for a safe working environment. This review paper provides fundamental knowledge on the hand biomechanics in terms of anthropometry, kinematics, kinetics, and EMG.

## 8 Summary and conclusion

### 8.1 Hand anthropometry

Hand anthropometry data can be used to design hand-guard products (e.g., gloves), hand-controlled products (e.g., remote control, mouse), and hand-operated tools (e.g., screwdriver, hammer). Hand anthropometry can be directly measured using various equipment and devices. In recent times, 3D scans are commonly used for this purpose because they can measure diverse hand areas precisely and easily. Hand anthropometry dimensions are largely divided into length, breadth, and circumference under the static condition. In general, the length and breadth have 50 and 10 variables, respectively. The circumference has 10 variables (Table 1). When using hand anthropometry data, choosing the appropriate dimensions and number of populations and individuals for the purpose of the study is very important [103]. Previous studies have failed to consider the breadth and circumference of the thumb as measurement dimensions. Thus, future research is required to measure the thumb dimensions.

### 8.2 Hand kinematics

For accurate evaluation of kinematic variables, various fields commonly use a 3D motion analysis system. This system can obtain 3D data more reliably compared with other methods [41]. The kinematic hand model and marker attachment methods require 3D motion analysis to evaluate the kinematic variables (Table 2). Many researchers have difficulties with selecting a marker attachment method to accurately measure hand functions. Based on this review, the “one marker per joint” method is recommended for greater patient comfort and easy marker placement, although any marker attachment method can be used under static conditions. The “three markers per segment” and “cluster marker” methods evaluate hand movements more accurately because of their robustness to skin movement. Thus, they are recommended for experiments conducted under dynamic conditions [104].

Table 3 lists the ROMs for finger flexion according to previous studies. The PIP joint (mean: 105°) has the largest flexion ROM followed by the MCP (mean: 84°) and DIP joints (mean: 69°). Table 4 lists the joint flexion angles for various hand functions. These previous studies focused mainly on the flexion angle of the four fingers excluding the thumb. However, the thumb is the most important part of the hand and has a wide range of activities during hand functions [105]. Thus, future research will involve examining the ROM and hand functions of the thumb, and the various hand functions will be measured in 3D.

### 8.3 Hand kinetics

The technologies for kinetics evaluation can be roughly divided into direct and indirect measurements. In general, the external load is measured directly with instruments, and the internal load is predicted analytically through kinetic models. Many previous studies have focused on measuring the force, moment, and torque during hand functions. Evaluating the joint force, moment, and torque requires accurate anthropometry data such as the segment mass, center of mass, center of gravity, and radius of gyration. Thus, accurate anthropometry data of the hand will be considered to develop a hand kinetics model in future research.

### 8.4 Hand EMG

In EMG, the most important factors are choosing suitable muscles, accurate attachment of the electrodes, and choosing a suitable signal-processing method for the research purpose. Table 6 lists the most commonly used hand muscles in hand functions when researchers use EMG. Most studies have used signal-processing methods such as RMS and MPF. Previous studies on muscle fatigue and characteristics based on the EMG signals have only considered static postures, not dynamic hand functions. They simply considered the relative muscle activity from the MVC. However, in the case of dynamic hand functions, the muscle fiber depth and length change with time and distance, and therefore, changes occur between the sEMG electrode and muscle fiber. Moreover, a muscle–tendon moment difference is generated when the muscle contraction velocity changes, and rapid motor unit recruitment by contraction shows flexible signal characteristics [102]. Therefore, the EMG signal of dynamic hand functions should be interpreted differently compared with that of static hand functions.

## Conflicts of interest

All contributing authors declare no conflicts of interest.

## Figures and Tables

**Table 1:** Summary of hand anthropometry dimensions

Length				Breadth/circumference	
No.	Variable	No.	Variable	No.	Variable
1	D1 length	26	D2 MCP link length	51	D2 PIP joint breadth
2	D2 length	27	D3 MCP l link length	52	D3 PIP joint breadth
3	D3 length	28	D4 MCP link length	53	D4 PIP joint breadth
4	D4 length	29	D5 MCP link length	54	D5 DIP joint breadth
5	D5 length	30	D1 PIP link length	55	D2 DIP joint breadth
6	Crotch 1 height	31	D1 DIP link length	56	D3 DIP joint breadth
7	Crotch 2 height	32	D2 DIP link length	57	D4 DIP joint breadth
8	Crotch 3 height	33	D2 MCP link length	58	D5 DIP joint breadth
9	Crotch 4 height	34	D2 PIP link length	59	Hand breadth from digitizer
10	D1 height	35	D3 DIP link length	60	D1 IP joint breadth
11	D2 height	36	D3 MCP link length	61	Hand breadth
12	D3 height	37	D3 PIP link length	62	D2 PIP joint circumference
13	D4 height	38	D4 DIP link length	63	D3 PIP joint circumference
14	D5 height	39	D4 MCP link length	64	D4 PIP joint circumference
15	D1 tip to wrist crease length	40	D4 PIP link length	65	D5 PIP joint circumference
16	D2 tip to wrist crease length	41	D5 DIP link length	66	D2 DIP joint circumference
17	D3 tip to wrist crease length	42	D5 MCP link length	67	D3 DIP joint circumference
18	D4 tip to wrist crease length	43	D5 PIP link length	68	D4 DIP joint circumference
19	D5 tip to wrist crease length	44	Palm length	69	D5 DIP joint circumference
20	D1 link length	45	Hand length	70	D1 IP joint circumference
21	D2 link length	46	Wrist-index grip length	71	Hand circumference
22	D3 link length	47	Wrist-thumbtip length		
23	D4 link length	48	Forearm-hand length		
24	D5 link length	49	Hand length from digitizer		
25	D1 MCP link length	50	Thumbtip reach		

D1, Digit 1 (thumb), D2, Digit 2 (index finger), D3, Digit 3 (middle finger), D4, Digit 4 (ring finger), D5, Digit 5 (little finger), MCP, metacarpophalangeal, PIP, proximal interphalangeal, DIP, distal interphalangeal, IP: interphalangeal.

**Table 2:** Marker attachment method and kinematic model of previous studies

Model	Attachment method	Authors
Cheng and Pearcy's model	One marker ∗	Gupta et al [46], Carpinella et al [49], Baker et al [51], Bazański [63]
	Two markers †	Ryu et al [52], Chiu et al [53], Sakai et al [54]
Eulerian angle model	Three markers ‡	Buczek et al [56], Cerveri et al [57]
	Cluster marker	Degeorges et al [58], Gehrmann et al [59]

∗ One marker per joint

† Two markers per segment.

‡ Three markers per segment.

**Table 3:** Range of motion of finger flexion

Finger		Swanson [67]	Becker and Thakor [68]	Chao et al [25]	Hume et al [65]	Degeorges and Oberlin [69]	Yoshida et al [36]	Zheng and Li [70]	Mean (standard deviation)
Thumb	CMC	—	—	52.9	—	—	—	45	49 (6)
	MCP	—	—	—	56	—	77	50	61 (14)
	IP	—	—	—	—	—	81	80	81 (1)
Index	MCP	62	71	83	—	97	—	85	80 (13)
	PIP	—	104	101	—	110	—	100	104 (5)
	DIP	—	61	73	—	57	—	80	68 (11)
Middle	MCP	64	85	90	—	100	—	—	85 (15)
	PIP	—	104	103	—	114	—	—	107 (6)
	DIP	—	74	80	—	57	—	—	70 (12)
Ring	MCP	67	85	88	—	107	—	—	87 (16)
	PIP	—	107	105	—	110	—	—	107 (3)
	DIP	—	67	75	—	57	—	—	66 (9)
Little	MCP	64	86	90	—	105	—	—	86 (17)
	PIP	—	99	103	—	111	—	—	104 (6)
	DIP	—	71	78	—	58	—	—	69 (10)

Range of motion are presented in degrees. CMC, carpometacarpal joint; DIP, distal interphalangeal joint; IP, interphalangeal joint; MCP, metacarpophalangeal joint; PIP, proximal interphalangeal joint.

**Table 4:** Joint flexion angles for various hand functions (Chao et al., 1989)

Hand function	MCP	PIP	DIP
Tip pinch	48	50	25
Key pinch	20	35	20
Pulp pinch	48	50	0
Power grasp	62	48	23
Abduction	0	0	0
Adduction	0	0	0
Flexion	0	0	0
Extension	45	0	0
Briefcase grip	23	72	55
Holding glass	5	48	20
Opening big jar	50	55	35

Flexion angles are presented in degrees. DIP, distal interphalangeal joint; MCP, metacarpophalangeal joint; PIP, proximal interphalangeal joint. *Note.* From “Biomechanics of the hand,” by E.Y. Chao, K.N. An, W.P. Cooney, R.L. Linscheid, 1989. Hackensack (NJ): World Scientific; 1989. Reprinted with permission.

**Table 5:** Tendon and joint forces during various hand functions

Hand function	Finger	Tendon force		Joint force			Authors
		FDP	FDS	MCP	PIP	DIP	
Power grasp	—	4.0–20.0	1.25–15.0	—	—	—	Bright and Urbaniak [89]
	—	4.0	0.60	—	—	—	Schuind et al [88]
	Index	2.77	2.53	12.7	4.35	0.09	Chao et al [60]
	Middle	3.05	4.23	3.90	7.11	0.17	
	Little	3.37	3.40	4.50	6.02	3.31	
	Middle	3.37	3.75	5.18	6.80	3.89	Chao et al [25]
	Index	3.17–3.47	1.51–2.14	3.20–3.70	4.50–5.30	2.80–3.40	An et al [78]
Pinch grip	Index	—	—	5.50	4.60	—	Berme et al [90]
Tip pinch	—	2.50–12.5	1.00–7.50	—	—	—	Bright and Urbaniak [89]
	—	8.30	1.90	—	—	—	Schuind et al [88]
	Index	—	—	3.50–3.90	4.40–4.90	2.40–2.70	An et al [78]
Key pinch	Index			14.70–27.10	4.90–19.40	2.90–12.50	An et al [78]
Pulp pinch	—	—	—	4.00–4.60	4.80–5.80	3.00–4.60	An et al [78]

The forces are presented in Newton. DIP, distal interphalangeal joint; FDP, flexor digitorum profundus; FDS, flexor digitorum superficialis; MCP: metacarpophalangeal joint; PIP, proximal interphalangeal joint.

**Table 6:** Hand muscles and the action, origin, insertion, and location of common extrinsic muscles of hand functions

Muscle	Action	Origin	Insertion	Location
FDS	Flexion of PIP and MCP joints	Common tendon from the medial epicondyle of the humerus, coronoid process of the ulna, and oblique line of the radius	All of these tendons are inserted in the volar surface of the 2^nd^ phalanx	Point index finger to biceps tendon and insert needle electrode from the ulna to the tip of the index finger. The electrode travels through the palmaris longus
FDP	Flexion of DIP joints	Upper three-fourths of volar and medial surfaces of the ulna and interosseous membrane	Volar surfaces of bases of distal phalanges of the 4 fingers	Place the tip of the little finger on the olecranon and the ring, middle, and index fingers along the shaft of the ulna
FPL	Flexion of IP and MCP joints of the thumb	Medial epicondyle of the humerus	Palmar aponeurosis and flexor retinaculum	At the junction of the upper and middle third of a line joining the medial epicondyle and middle of the volar surface of the wrist
EPL	Extension of IP and MCP joints	Ulna adjacent to the interosseous membrane	Dorsal base of the thumb Distal phalanx through the thumb extensor mechanism	On the dorsal side of the forearm
EPB	Extension of MCP joint of the thumb	Radius adjacent to the interosseous membrane	Over tendons of radial extensors and brachioradialis to the base of the proximal phalanx of the thumb	On the dorsal side of the forearm
ED	Extension of MCP joints	Common extensor tendon from the lateral epicondyle of the humerus	On the dorsal surface of the base of the second to 5^th^ phalanges of the fingers	Grasp the forearm at the junction of the upper and middle third with the thumb and middle finger on the radius and ulna. With the index finger, bisect these 2 points and insert a needle electrode at the tip of the index finger to a depth of 1.27 cm
APL	Abduction of the thumb	From dorsal surface of the body of the ulna, interosseous membrane, and middle third of the body of the radius	Lateral aspect of the base of the 1^st^ metacarpal	Over the shaft of the radius at the mid forearm. The electrode travels through the ED

DIP, distal interphalangeal joint; ED, extensor digitorum communis; EPB, extensor pollicis brevis; EPL, extensor pollicis longus; FDP, flexor digitorum profundus; FDS, flexor digitorum superficialis; FPL, flexor pollicis longus; IP, interphalangeal joint; MCP, metacarpophalangeal joint; PIP, proximal interphalangeal joint.
